# Spontaneous breathing approach in mild congenital diaphragmatic hernia: A resuscitation algorithm

**DOI:** 10.3389/fped.2022.945090

**Published:** 2022-07-18

**Authors:** Emily J. J. Horn-Oudshoorn, Ronny Knol, Suzan C. M. Cochius-den Otter, Arjan B. te Pas, Stuart B. Hooper, Calum T. Roberts, Neysan Rafat, Thomas Schaible, Willem P. de Boode, Robin van der Lee, Anne Debeer, Florian Kipfmueller, Charles C. Roehr, Irwin K. M. Reiss, Philip L. J. DeKoninck

**Affiliations:** ^1^Division of Neonatology, Department of Paediatrics, Erasmus MC University Medical Center, Rotterdam, Netherlands; ^2^Intensive Care and Department of Paediatric Surgery, Erasmus MC University Medical Center, Rotterdam, Netherlands; ^3^Division of Neonatology, Department of Paediatrics, Leiden University Medical Center, Leiden, Netherlands; ^4^The Ritchie Centre, Hudson Institute for Medical Research, Monash University, Melbourne, VIC, Australia; ^5^Department of Paediatrics, Monash University, Melbourne, VIC, Australia; ^6^Department of Neonatology, University Medical Center Mannheim, Mannheim, Germany; ^7^Division of Neonatology, Department of Paediatrics, Radboud Institute for Health Sciences, Radboudumc Amalia Children's Hospital, Nijmegen, Netherlands; ^8^Department of Neonatology, University Hospitals Leuven, Leuven, Belgium; ^9^Department of Neonatology and Paediatric Critical Care Medicine, University of Bonn Children's Hospital, Bonn, Germany; ^10^National Perinatal Epidemiology Unit, Medical Sciences Division, Nuffield Department of Population Health, University of Oxford, Oxford, United Kingdom; ^11^Newborn Services Southmead Hospital, North Bristol Trust, Bristol, United Kingdom; ^12^Faculty of Health Sciences, University of Bristol, Bristol, United Kingdom; ^13^Division of Obstetrics and Fetal Medicine, Department of Obstetrics and Gynaecology, Erasmus MC University Medical Center, Rotterdam, Netherlands

**Keywords:** congenital diaphragmatic hernia, intubation, spontaneous breathing approach, neonatal resuscitation, birth

## Abstract

**Background:**

Infants with a congenital diaphragmatic hernia (CDH) and expected mild pulmonary hypoplasia have an estimated survival rate of 90%. Current guidelines for delivery room management do not consider the individual patient's disease severity, but an individualized approach with spontaneous breathing instead of routine mechanical ventilation could be beneficial for the mildest cases. We developed a resuscitation algorithm for this individualized approach serving two purposes: improving the success rate by structuring the approach and providing a guideline for other centers.

**Methods:**

An initial algorithm was discussed with all local stakeholders. Afterwards, the resulting algorithm was refined using input from international experts.

**Results:**

Eligible CDH infants: left-sided defect, observed to expected lung-to-head ratio ≥50%, gestational age at birth ≥37.0 weeks, and no major associated structural or genetic abnormalities. To facilitate fetal-to-neonatal transition, we propose to start stabilization with non-invasive respiratory support and to adjust this individually.

**Conclusions:**

Infants with mild CDH might benefit from an individualized approach for neonatal resuscitation. Herein, we present an algorithm that could serve as guidance for centers implementing this.

## Introduction

Around 70% of all infants with a congenital diaphragmatic hernia (CDH) are detected during prenatal screening (1–3). This provides an opportunity for early referral to specialized centers, additional diagnostic procedures, and individualized counseling. For isolated cases, postnatal outcomes largely depend on the extent of the pulmonary disease (4, 5). Antenatal ultrasound measurement of the contralateral lung, expressed as the observed to expected lung-to-head ratio (o/e LHR), is the most validated method to estimate the severity of pulmonary hypoplasia (4, 5). Liver position and defect-side are additional independent predictors of postnatal outcomes (3, 4, 6). Based on these parameters, one can distinguish a group with a relatively mild degree of pulmonary hypoplasia, corresponding with an estimated survival rate of 90% (4, 5). Current guidelines on delivery room management apply to all neonates with CDH and do not take the individual neonate's disease severity into account. An example of this is initial mechanical ventilation, which might be too aggressive for infants with expected mild pulmonary hypoplasia, given the favorable outcomes, the risk of ventilator-induced lung injury, and the stress caused by intubation (3–5, 7, 8). A more individualized approach has the potential to avoid overtreatment and risks of intubation.

The Erasmus MC implemented a trial of spontaneous breathing for a specific subset of infants (isolated left-sided CDH, o/e LHR ≥50%, and intra-abdominal liver position) in December 2014 (9). A retrospective single-center audit recently demonstrated that the spontaneous breathing approach (SBA) was feasible, but 60% of cases still required intubation in the first hours after birth (10). On the other hand, there was an apparent decrease in the total length of hospital stay in successful cases and, more importantly, there were no adverse effects of the delayed intubation in cases that failed the SBA (10). These results justify further evaluation of this approach. Yet, the low success rate in this small series highlights that optimal case selection is challenging and emphasizes the need for a standardized management algorithm (10). Meanwhile, other centers have already implemented the SBA or are interested. For these reasons, we developed a resuscitation algorithm that serves two purposes: improving the success rate by structuring the approach and providing a guideline for centers that consider implementation.

## Methods

Algorithm development was a two-step process: first, it was drafted and discussed by all stakeholders that are involved in the care of CDH infants in the Erasmus MC (i.e., neonatal nurses, neonatologists, obstetricians, pediatric intensivist, and pediatric surgeons); second, the resulting algorithm was optimized with input from international experts on neonatal resuscitation, CDH management, and fetal/neonatal physiology. Medical ethical approval for prospective data collection was obtained in the Erasmus MC as the initiating center (MEC-2021-0304) and will be obtained in all centers that start data collection.

## Results

### Patient selection

Only CDH infants with expected mild pulmonary hypoplasia are considered candidates. We propose the following eligibility criteria depicted in Table 1. We recommend discussing the initial ventilation strategy for each case during a multidisciplinary meeting around 30 weeks of gestation, involving all caregivers.

**Table 1:** Eligibility criteria for spontaneous breathing approach.

**Eligibility criteria**
- Left-sided defect (3, 11); - o/e LHR ≥50% (measured on ultrasound at 30 weeks of gestational age [28-32 weeks] *or* on initial visit in case of detection after 30 weeks of gestational age) and abdominal liver position (4, 5); - Gestational age at birth ≥37.0 weeks (12); - No antenatal diagnosed major associated structural or genetic abnormalities (13).

*o/e LHR, observed to expected lung-to-head ratio*.

### Clinical algorithm

**Figure 1:**
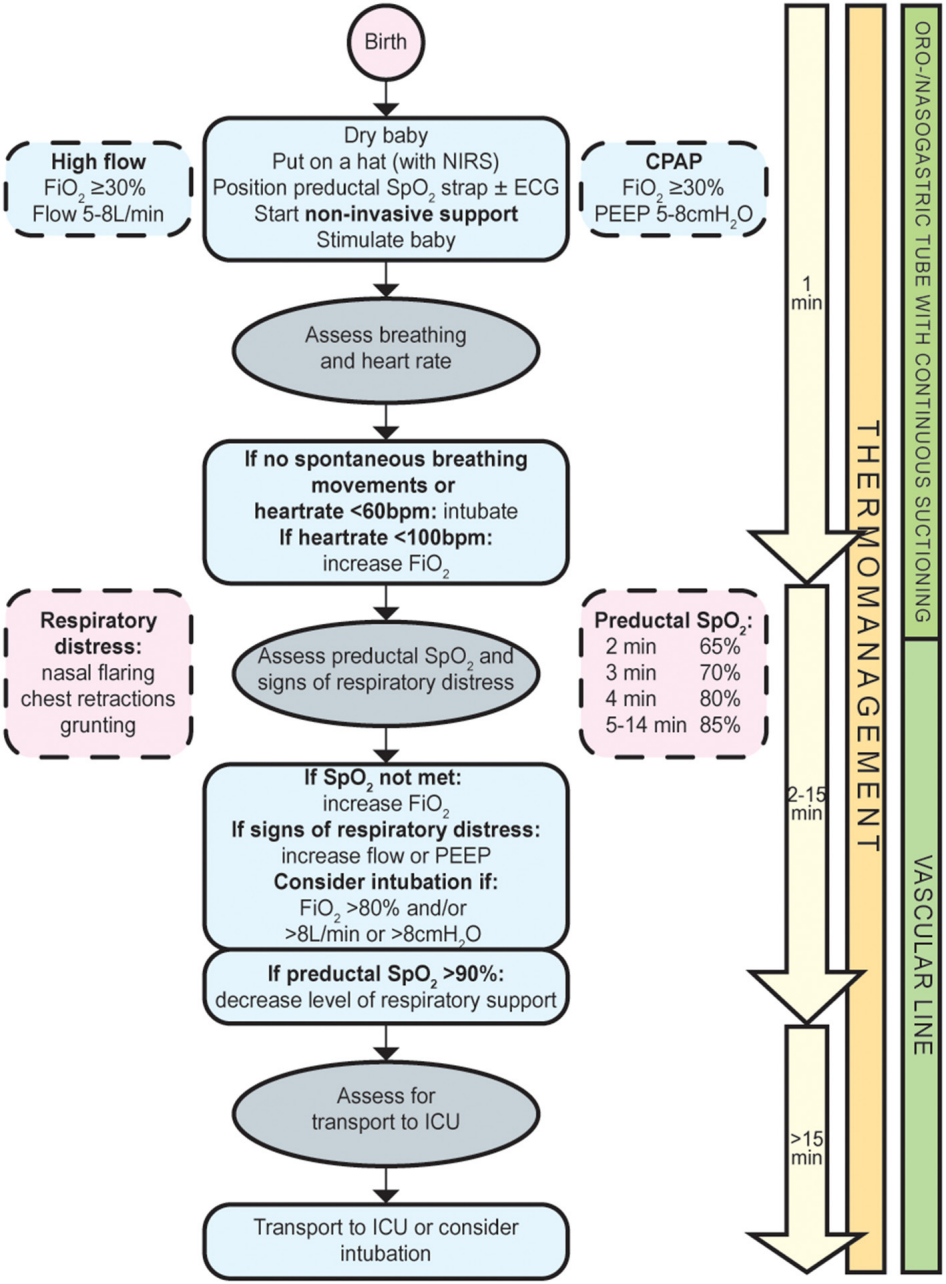
Flowchart spontaneous breathing approach for infants with a congenital diaphragmatic hernia.

The primary aim of perinatal stabilization of infants with a CDH is to establish adequate oxygenation whilst avoiding hypoxia, hyperoxia, and high peak airway pressures (9, 14). In the abovementioned series, reasons for intubation in the delivery room were low SpO_2_-levels, absence of breathing movements, or signs of respiratory distress (10). To facilitate the fetal-to-neonatal transition, and, thus, the success of the SBA, we suggest to start stabilization with non-invasive respiratory support and to adjust this individually (Figure 1). It is, however, not clear whether the fetal-to-neonatal transition in these infants is more favorably supported by additional FiO_2_ and/or continuous distending airway pressures (high flow or CPAP). To enable implementation in other centers, we leave it up to the centre's discretion to decide whether high flow or CPAP is more feasible within their local logistics and standard of care. To minimize the negative effects of potential abdominal distension associated with non-invasive respiratory support, we recommend early insertion of an oro-/nasogastric tube.

We recommend to:

- Initiate nasal high flow or CPAP and subsequently titrate up or down by continuously evaluating the infant's respiratory status using the European Neonatal Life Support guidelines (15);
- Consider intubation in case of insufficient spontaneous breathing movements, heartrate <60/min, FiO_2_ > 80%, flow >8 L/min, or CPAP > 8 cmH_2_O;
- Decrease the level of respiratory support if preductal SpO_2_ >90%;
- Insert an oro-/nasogastric tube with continuous suctioning.

## Discussion

This resuscitation algorithm presents an individualized approach for infants with a CDH and predicted mild pulmonary hypoplasia. We acknowledge that the proposed algorithm is based on expert-opinion and low-grade, single-center evidence (Scottish Intercollegiate Guidelines Network criteria, grade of recommendation D) (16). Ideally, this strategy should be tested in a randomized controlled trial. However, the lack of equipoise in centers that have already implemented the SBA would pose a challenge for reaching a sufficient sample size to evaluate the full extent of the various clinically relevant outcomes. Instead, prospective observational data collection of CDH infants cared for with the SBA is in progress within the framework of an international research consortium: the very mild CDH—SBA consortium (VeSBA). We share our algorithm, so that the SBA may be adopted by other centers and we invite their contribution to this prospective registry. We emphasize that strict adherence to the algorithm is not a prerequisite to join the VeSBA consortium and local adaptations are obviously acceptable.

## Conclusion

Current guidelines on delivery room management for infants with a CDH do not take into account the individual patient's disease severity. However, the spontaneous breathing approach is an individualized approach for infants with a relatively mild CDH that could prevent overtreatment in this specific subgroup.

## Data Availability Statement

The original contributions presented in the study are included in the article/supplementary material, further inquiries can be directed to the corresponding author.

## Ethics Statement

Medical ethical approval for prospective data collection within the international VeSBA registry was obtained in the Erasmus MC as the initiating centre (MEC-2021-0304) and will be obtained in all centres that start data collection. Informed consent for prospective data collection will be obtained for each patient cared for with the spontaneous breathing approach.

## Author Contributions

EH-O, RK, SC, IR, and PD were all involved in the conception of this manuscript and the design of the algorithm. AP, SH, CTR, NR, TS, WB, RL, AD, FK, and CCR contributed to the algorithm. EH-O drafted the initial manuscript, which was critically reviewed by RK, SC, AP, SH, CTR, NR, TS, WB, RL, AD, FK, CCR, IR, and PD. All authors have approved the final manuscript as submitted and agree to be accountable for all aspects of the work.

## Funding

EH-O and PD are supported by a grant from Sophia Children's Hospital Foundation (SSWO, grant S19-12).

## Conflict of interest

The authors declare that the research was conducted in the absence of any commercial or financial relationships that could be construed as a potential conflict of interest.

## Publisher's Note

All claims expressed in this article are solely those of the authors and do not necessarily represent those of their affiliated organizations, or those of the publisher, the editors and the reviewers. Any product that may be evaluated in this article, or claim that may be made by its manufacturer, is not guaranteed or endorsed by the publisher.

## Abbreviations

CDH: congenital diaphragmatic hernia
o/e LHR: observed to expected lung-to-head ratio
SBA: spontaneous breathing approach.
